# Effects of World War II on Infectious Diseases in the Persian Gulf

**DOI:** 10.34172/aim.28778

**Published:** 2024-05-14

**Authors:** Mohmmad Jafar Chamankar

**Affiliations:** ^1^History Department, University of Urmia, Urmia, Iran

**Keywords:** Health, Infectious diseases, Iran, Persian Gulf, Sea of Oman, World War II

## Abstract

With the allied invasion, the southern half of Iran became the scene of a large presence of British and American occupation forces. The negative consequences of the styling of foreign elements during all the years of war and even afterward affected these areas in various dimensions of their health. The negative consequences of the occupation of southern Iran, the health and healthcare system of this part of Iran suffered problems in various forms of shortage of medicines, equipment, and treatment staff, especially the spread of various infectious and communicable diseases. The article aims to examine the effects of World War II on the southern, southwestern, and eastern regions of Iran from 1939 to 1945 and its consequences in the spread of infectious diseases in these regions. The research with an analytical-historical method relies on the library method and is based on the study of the data of unpublished documents from the archives of the National Archives and Library Organization, medical and economic social publications, and various local and public newspapers of Iran during this period. The study of documents and publications shows that due to Allied restrictive policies and successive waves of famine and widespread malnutrition, epidemic diseases, and drug monopoly, these areas experienced a period of severe decline in public health and spread of various infectious diseases.

## Introduction

 On the morning of August 25, 1941, in violation of neutrality, Iran was attacked in the north and south by the Soviets and the United Kingdom. As a result, the southern half of Iran was captured by the British and then American forces, and during all the years of war, their heavy presence was seen in these areas. With the Allied occupation of Iran, public health in various forms throughout the southern half of the country was affected by its negative consequences. A combination of poverty, famine, severe food shortages, impossibility of adhering to health and public health necessities, spread of various infectious diseases, and the social consequences and negative economic consequences of the presence of thousands of Allied forces and Polish migrants, had a profound and complex impact on the health of the islands and parts of the northern Persian Gulf and the sea of Oman and its post-coastal areas, which continued to exert its effects for a long time after the end of the war. The research method in this historical and analytical paper is based on the library method. Historical evidence has been used in several categories of books, archival documents, and especially the press. Newspapers and magazines are important in this regard, which in a way include eyewitness reports. These reports were recorded by people who participated in the historical events of this period. As such, various publications were examined from 1939 to 1946 daily and in a long time process. Based on the descriptive-analytical method of the research, as well as the study of books, documentary data, and especially publications, the author tries to answer the following question: how did the occupation of Iran exacerbate the spread of infectious diseases in the northern regions of the Persian Gulf and the sea of Oman. The research hypothesis is based on the principle that a set of complementary areas of malfunction resulting from the widespread presence of occupying elements, the sanitary conditions of the islands and ports and backwaters of southern Iran were critical to the pre-war period, which also showed negative consequences until the years after the end of the war.

 As far as the author knows, despite valuable research on the history of Iranian medicine and the economic consequences of World War II on the Persian Gulf, there is no specific mention of the effects and consequences of the Second International War on the health of the southern regions of Iran. The cause is a severe shortage of resources, documentation, and statistical data. As a result, this effort could supply the necessity of doing research and serve only as the beginning of more comprehensive research. Local newspapers such as *Kushesh*, published by Shukrullah Safavi, the representative of Bushehr in the parliament, *Estakhr*, *Sepantā*, *Pars*, *Naqhsh -e jahān*, and *Gulastan* are of great importance in this process. In fact, in many cases, local publications are the only sources that provide the researcher with data on health developments at this point. The country’s public publications, including *Ettelāāt*, are especially important in reflecting the views of the National Assembly representatives from the southern regions and reports of health developments in these regions. Economic publications of this period also convey valuable information to researchers. Specialized publications related to the health sector also contain materials with a more scientific approach, focusing on the effects of World War II on the public health of islands and ports. The Chronicles of this era, including *Sālnāme ‌ ye donyā*, have also provided important data to the researcher in this field.

## Discussion

###  Shortages and Drug Seizures and Their Impact on the Decline of Iran’s Health System

 With the outbreak of World War II in 1939, the arrival of foreign specialized medicines in Iran faced a serious problem.^1^ As a result, drug shortages were one of the most important problems of the Iranian Health System during World War II.^2^ Medicines were rare or expensive,^3^ the health situation in most villages and southern cities was very bad and the hospital equipment was insufficient from head to head in the country.^4^ Medicinal products in 1943 and 1944, were scarce and the price increased in increasing Form.^5^ In the port of Bushehr, half of the population was infected with malaria.^6,7^ which the health centers did not have enough medicine to treat, especially the pills Quinine and Cinchona.^8^ In 1943, the shortage of medicines reached the point where, for example, the price of one ampoule, which was once 1 shilling, reached 1 lira and 5 shillings, and a box of tablets, which was previously 3 shillings, reached 1 and a half lira. A pill, usually priced at 1 Pence, was sold for 1 shilling and even more and was often not “obtained” due to scarcity.^9^ The shortage of medicines and health problems exacerbated Iran’s monopoly and illegal trade in medical supplies.^10-12^ It was based on this that Amir Timur, a member of Parliament, in March 1941, referring to the confiscation of Medicine by “a few low-nature people”, said: “The medicine that was yesterday 1 Quran, today will not be found to 4 tomans”.^13,14^ The issue of the lucrative drug trade was even seen among state officials.^15^ In the summer of 1945, Radiology film became extremely rare, its price increased by 10%, leaving patients with the “most difficult problems.^16^ With the increase in the smuggling of medical supplies, the Department for the Prohibition of Drug Possession was formed in the quarter of 1942 under the chairmanship of Dr. Taghi Majlisi.^17^ The ban on the issuance of medicines from March 1941 and the facilitation of imports and clearance from customs were other ways to combat drug trafficking in Iran.^18^

###  Decline in Public Health 

 With the Allied occupation of Iran, public health across the country was affected by its negative consequences.^19^ Like other major wars, deaths from various diseases that sometimes outweighed the direct casualties of the conflicts themselves became the cause of Iran.^20^ With the influence of the sinister effects of the war in Iran and the emergence of famine, diseases caused by poverty and malnutrition gradually began and spread.^21^ The sharp decline in quality of life limited medical care in large cities and even the capital.^22^ The use of unhealthy drinking water, which flowed mainly from canals flowing in different areas of cities, played an important role in the spread of infectious diseases. In most cities, drinking water sewage and alley waste flowed in a duct, and “the toilets” were located near water wells.^23,24^ An American who traveled to Tehran in 1945، mentions the widespread poverty that attracts the attention of every viewer and the pathogenic water channels that “can hardly be called Water”.^25^ John Scleese Avery, the son of Dr Avery, an adviser to the Ministry of Health who arrived in Iran in 1945 has pointed to a lack of Water Sanitation:

 “How they didn’t die from such a life with minimal hygiene was a surprise to me”.^26^

 In the booklet “General Information of Iran”, which was given to American soldiers living in Iran during World War II, it is stated that in Iran, sanitary drinking water is not found and this water is full of germs.^27^

###  Outbreaks of Communicable Diseases

 The presence of thousands of foreign troops in different regions of Iran, whose logistics were mainly carried out by domestic products, along with economic disruptions, especially in agricultural production, increased economic problems and severe food shortages.^28^ Gradually, famine swept across the southern coast of Iran, causing widespread human casualties.^29^ It was based on this that the newspaper “Today’s World” reported on malnutrition in these areas, stating that in the South, people use the blood of sheep and desert plants.^30^ As a result of absolute hunger, cases of cannibalism from human corpses have been reported in these areas.^31^ Accordingly, the unsanitary situation in the southern and southeastern pages of Iran was more severe. On the beaches, as a result of the lack of “Hygiene and dirty living”, and the lack of medicines and doctors, the number of deaths was always higher than in other regions of Iran.^32^ The ports of Bushehr, Borazjan, Dashtestan, and Port Kangan have been in poor sanitary conditions since 1939.^33^ The plague and its outbreak were always associated with heavy casualties.^34^ Smallpox was one of the diseases that caused widespread casualties in the governorate of Bushehr.^35^ According to the 7th Persian Army, smallpox was widely reported in 1940 in the Bushehr region, especially in Borazjan and dashtestan, Ahram, the Port of Genaweh, and the Port of Rig. Also, due to the plague in Jarom and Fars, the likelihood of the spread of this dangerous disease in Bushehr was high.^36^ In 1944-1945 the disease affected the city of Bushehr and the village of Bandargah. Authorities called for “urgent” assistance from the government due to a lack of doctors and medicines.^37^

 The Western states of Saudi Arabia, the eastern shores of the Red Sea, Iraq, and especially India, were the main focuses of the plague.^38^ The cholera wiped out more than 500 000 people in India from 1918 to 1941. The arrival of pilgrims, merchants, and Indian soldiers in Iran would have made the communicable disease more prevalent.^39^ Malaria was also highly susceptible to outbreaks around the coasts and backwaters of the Persian Gulf and the Sea of Oman.^40^ The presence of unsanitary reservoirs in these areas, which provided most of the people’s drinking water, added to the extent of the disease.^41^ Anopheles or malaria mosquitoes lay eggs in the water of swamps, rivers, and streams, and for some time the mosquito’s sperm and fetus lived in the water.^42^ Drinking swampy water, the Malaria bacillus entered the bodies of people and was” ruthlessly “ wasting a large group of villagers, “turning away” the rest and destroying an area.^43^ The extent of the outbreak was so widespread that whenever Medicine examined a disease that had fever and chills, it thought of malaria before “everything”.^44^

 Thus, the negative results of the allied presence brought the health and nutrition systems of the southern and southeastern regions of Iran to a critical stage.

## Conclusion

 With the violation of neutrality in August 1941 and the fall of Reza Shah, Iran went through a turbulent period of occupation, the negative consequences of the political transition phase, and the lack of military and economic concentration. To send aid to the Soviet Union, all the facilities of Iran, especially on the shores of the Persian Gulf and the Sea of Oman, were provided to the Allied forces. This was the widespread presence of foreign elements in Persia, Khuzestan, Bushehr, Hormuzgan, and the shores of the Sea of Oman and the aftermath of these regions and their all-round dominance over the bulk of the interactions in this area. With the Allied occupation of Iran, public health across the country was affected by its negative consequences. With the outbreak of World War II and then the occupation of Iran, the arrival of foreign specialized medicines faced a serious problem. The financial crisis and all-around lack of food caused the aggravation of poverty, severe malnutrition, rapid deterioration of the quality of life, and lack of attention to personal health care. Unhealthy drinking water, illegal trade in medicines and medical supplies, the spread of infectious diseases including smallpox, typhus, typhoid, cholera, skin and hair diseases in the absence of detergents and clean clothes and sanitary baths, the prevalence of eye diseases, especially trachea, which put children’s vision at serious risk, the spread of infectious diseases and the upward trend of mortality among children, the short-term and fatal consequences of the structural collapse of Health and medical care in Iran and its southern half were during the World War. These factors caused a kind of severe decline in health quality and access to health facilities compared to the pre-war period, in the era of the first Pahlavi rule.
