# Correction: Effects of World War II on Infectious Diseases in the Persian Gulf

**DOI:** 10.34172/aim.33705

**Published:** 2025-01-01

**Authors:** Mohammad Jafar Chamankar

**Affiliations:** ^1^History Department, Urmia University, Urmia, Iran

 This notice corrects the article titled “Effects of World War II on Infectious Diseases in the Persian Gulf,” published in 2024; 27(7):403-406 (DOI: 10.34172/aim.28778).

 In the main article and the author’s affiliation section, the address “History Department, University of Urmia, Urmia, Iran” has been updated to “History Department, Urmia University, Urmia, Iran.”

 This correction has been implemented in both the PDF and HTML versions of the article.

